# Hypothyroidism Presenting as Adynamic Ileus Mimicking a Mechanical Small Bowel Obstruction: A Diagnostic and Management Dilemma

**DOI:** 10.7759/cureus.50799

**Published:** 2023-12-19

**Authors:** Kacper Kubiszewski, Rachel W Chapman, Yelena Piazza, Dhruv Patel, Vladimir Neychev

**Affiliations:** 1 Medical School, University of Central Florida College of Medicine, Orlando, USA; 2 Pathology, University of Central Florida College of Medicine, Orlando, USA; 3 Pathology, UCF-Lake Nona Hospital, Orlando, USA; 4 Radiology, University of Central Florida College of Medicine, Orlando, USA; 5 Radiology, UCF-Lake Nona Hospital, Orlando, USA; 6 Surgery, University of Central Florida College of Medicine, Orlando, USA; 7 Surgery, UCF-Lake Nona Hospital, Orlando, USA

**Keywords:** hashimoto’s thyroiditis, intestinal pseudo-obstruction, adynamic ileus, hypothyroidism, small bowel obstruction

## Abstract

Patients who present with nausea, vomiting, constipation, and abdominal pain typically undergo workups for small bowel obstruction (SBO). SBO is commonly caused by mechanical obstruction due to adhesions, inflammatory conditions, or malignancies. Hypothyroidism is primarily associated with decreased basal metabolic rate and rarely, in severe cases, gastrointestinal motility dysfunction. We report a case of a 44-year-old man who presented to the emergency department with abdominal pain, nausea, and vomiting. The workup, including computed tomography, showed a small bowel feces sign, highly suspicious for a mechanical SBO. His past medical history was significant for a poorly controlled hypothyroidism due to Hashimoto’s thyroiditis with a markedly elevated thyroid stimulating hormone (TSH) level. He had no prior surgical history, and his family history was significant for a suspected inflammatory bowel disease (IBD) in his son. The patient failed initial resuscitative nonoperative management and underwent exploratory laparoscopy that revealed diffusely dilated small bowel loops with no obvious cause of mechanical obstruction. Inflammatory markers for IBD were found to be negative, and the patient’s gastrointestinal motility gradually improved with daily intravenous levothyroxine.

## Introduction

Small bowel obstruction (SBO) causes approximately 30,000 deaths annually in the United States [1]. The most common causes of mechanical SBO are adhesions, hernias, inflammatory conditions such as Crohn’s disease, and neoplasms [1,2]. Other conditions leading to functional bowel dysmotility disorders may lead to adynamic ileus and pseudo-obstruction mimicking mechanical SBO [1].

Affecting approximately one in 300 people in the United States, hypothyroidism is generally associated with systemic symptoms of decreased basal metabolic rate, weight gain, cold intolerance, dry skin, hair thinning, depression, and fatigue [3]. Symptoms of decreased gut motility tend to be a mild component of hypothyroidism, and adequate thyroid hormone replacement therapy has been shown to alleviate these symptoms [4]. Severe gastrointestinal (GI) dysmotility, causing SBO-like clinical and radiological features, is an extremely rare hypothyroidism presentation, particularly in the absence of the usual systemic symptoms of hypothyroidism. We present a case of a 44-year-old man with uncontrolled hypothyroidism who presented with findings similar to those of mechanical SBO that gradually resolved following the titration of his daily levothyroxine dosage.

## Case presentation

A 44-year-old man presented to the emergency department with nausea, vomiting, and worsening postprandial abdominal discomfort for the past day. He stated he had vomited several times and had not had a bowel movement or gas for 48 hours. The patient denied chest pain, shortness of breath, fevers, chills, or recent illnesses. He also denied fatigue, lethargy, cold intolerance, weight gain, change in voice, and dry skin. His medical history consisted of hypothyroidism and dyslipidemia, and his home medications included levothyroxine 125 mcg once every morning and atorvastatin 40 mg once every morning. However, the patient reported taking levothyroxine irregularly for the last several months. The patient denied any prior surgeries. He also denied any alcohol, tobacco product, or recreational drug use. His family history was significant for a son with suspected inflammatory bowel disease (IBD).

An initial physical exam revealed a well-appearing patient in no acute distress. His temperature was 98.4 Fahrenheit, his pulse was 71 beats per minute, his respiratory rate was 18 breaths a minute, his blood pressure was 141/89 mmHg, and his oxygen saturation was 100% on room air. Abdominal examination revealed normoactive bowel sounds and diffuse abdominal tenderness to palpation but no focal tenderness. No guarding, rebound tenderness, or abdominal masses were noted.

The patient’s initial comprehensive metabolic panel was remarkable for creatinine of 1.4 mg/dL. His complete blood count (CBC) revealed white blood cells (WBCs) of 8.3x10^3^/uL, hemoglobin of 16.5 g/dL, and hematocrit of 50.5%. Urinalysis revealed 1+ urine protein, 2+ urine ketones, trace blood, 1+ bilirubin, 2-5 urine RBCs, and 0-2 urine WBCs, and was otherwise unremarkable. His thyroid stimulating hormone (TSH) was 25.45 mLU/L, an increase from 5.5 mLU/L from approximately one month prior to his presentation.

**Figure 1:**
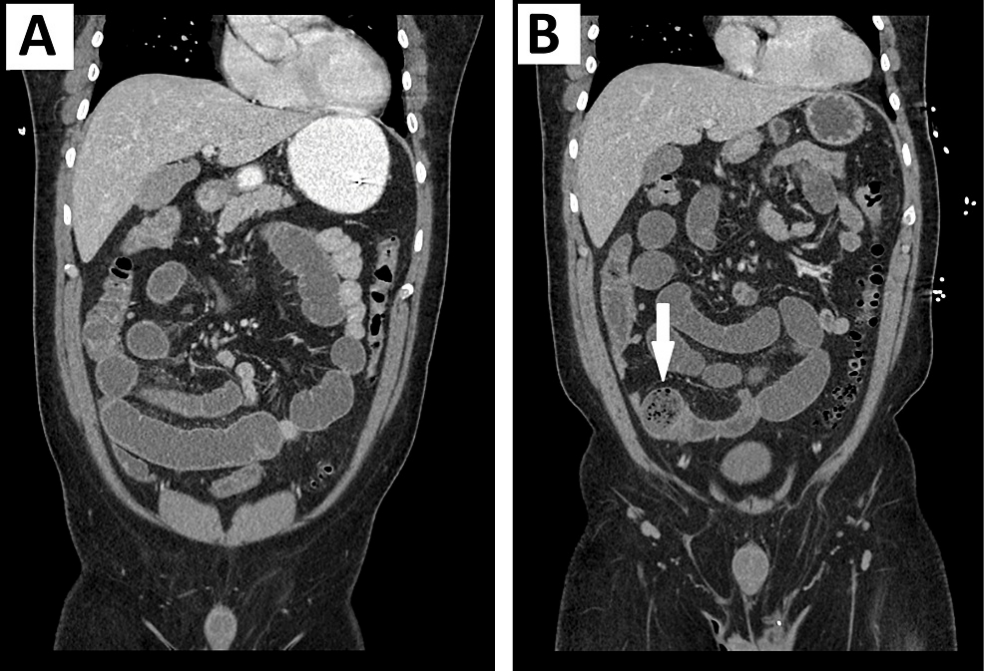
Preoperative CT scan findings A) Representative coronal image of the CT scan of the abdomen and pelvis with dilated small bowel loops suspicious for SBO; B) Representative coronal image with dilated small bowel loops and feces sign (arrow)

A computed tomography (CT) of the abdomen and pelvis with intravenous contrast revealed dilated small bowel loops with a transition point in the right lower quadrant. A feces sign was found in the mid to distal ileum with a distended proximal small bowel (Figures 1A, 1B). These radiological findings in tandem with the clinical presentation were highly suspicious for a mechanical SBO. Nonoperative management was attempted first. A nasogastric tube was inserted and placed on low-intermittent suction. Piperacillin/tazobactam was started at 3.375 g and given every eight hours. Maintenance IV fluids, IV pain medications, and IV antiemetics were initiated as well. He was also started on levothyroxine 125 mcg, IV every 24 hours to be given in the mornings. A repeat CT scan with oral contrast was scheduled for the following day.

The following morning, the patient experienced further discomfort and distress after attempting to drink the oral contrast, which he could not finish. Because of this, the decision was made to proceed with exploratory laparoscopy to determine the etiology of his obstruction. Intraoperatively, he was noted to have multiple foci of anteriorly creeping fat in the ileum and distal jejunum, and a non-obstructing inter-mesenteric fat bend in the middle third of the ileum that was removed. There was a mesenteric induration suspected to be a reactive lymph node just proximal to the inter mesenteric fatty bend that was biopsied. There was also a counter-mesenteric fat pad on the anterior jejunal wall measuring approximately 5 cm long and fine non-obstructing adhesions of the terminal ileum to the pelvic floor. His bowel loops were mildly to moderately dilated, but there was no obvious transition point or site of obstruction. A Jackson-Pratt drain was placed in the right lower quadrant.

**Figure 2:**
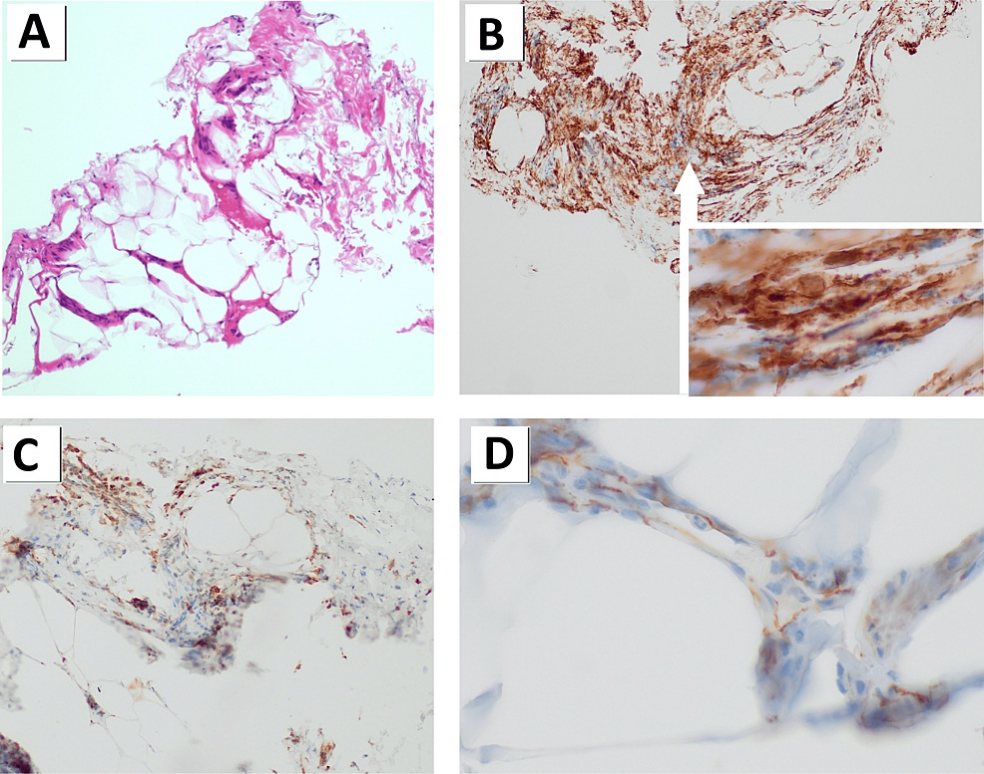
Hematoxylin-eosin and immunohistochemical staining of the intraoperative biopsy specimens A) Representative image (x100) of hematoxylin-eosin staining of mesenteric fibroadipose tissue with pericellular fibrosis and septal fibrosis; B) Representative image (x100) immunohistochemical staining of CD34 with the right lower corner insert depicting a proliferation of spindle-shaped fibroblasts and myofibroblastic differentiation (x400); C) Representative image (x100) of CD68 with histiocytic proliferation without granulomatous formation; D) Representative image (x400) of beta-catenin with membranous but not nuclear reactivity that rules out desmoid-type fibromatosis.

The patient’s intraoperative findings were suspicious for creeping fat, and a family history of possible IBD warranted investigation for possible Crohn’s disease, but both p-ANCA and saccharomyces cerevisiae antibodies were within normal limits. His biopsy results found no evidence of granulomas or histiocytes. Microscopic examination and hematoxylin-eosin staining revealed small fragments of fibroadipose tissue with fragments of vascular wall segments and foci of pericellular and interlobular fibrosis that exhibited mild-to-moderate patchy lymphohistiocytic and fibroblastic proliferation (Figure 2A). Immunohistochemical staining demonstrated spindle cells of myofibroblastic differentiation positive for CD34 and negative for beta-catenin (Figures 2A-2C). CD68-positive macrophages were present but no epithelioid histiocytes or formation of granulomas were identified (Figure 2D). Further workup with colonoscopy was delayed.

A radiograph of the small bowel with contrast was obtained two days postoperatively. No sign of obstruction was noted. Contrast was present in the right colon 180 minutes after ingestion, and his imaging findings were consistent with adynamic ileus.

Two days after surgery, TSH was repeated and found to be 41.14 mLU/L (increased from 25.45 mLU/L). The IV levothyroxine dose was increased to 175 mcg daily every morning. Daily trending of TSH was found to be 23.70 mLU/L, 36.20 mLU/L, 18.81 mLU/L, and 25.17 mLU/L on postoperative days three, four, five, and six, respectively. The levothyroxine dose was increased to 225 mcg, IV daily every morning. The patient’s condition and bowel dysmotility gradually improved. He was discharged home on postoperative day six with instructions to follow up with his endocrinologist and surgeon.

## Discussion

Approximately 70% of bowel obstruction cases are attributable to adhesions, commonly caused by prior abdominal or pelvic surgery [5,6]. Obstructive inflammation, hernias, and malignancies account for the remainder of cases, particularly in patients with no prior abdominal surgeries, as in this patient’s case [2,6,7]. A small fraction of SBO cases are caused by functional etiologies, such as adynamic ileus, narcotic bowel, acute colonic pseudo-obstruction, and acute mesenteric ischemia, which typically occur in the postoperative period or in association with underlying medical disorders (such as metabolic disturbances) [1,8,9].

Undiagnosed or untreated hypothyroidism is commonly marked by weight gain, cold intolerance, and fatigue [3]. In addition to decreased metabolic functions, hypothyroidism is biochemically characterized by the accumulation of glycosaminoglycans in the tissues resulting in interstitial edema of the skin and muscle, known as myxedema. Infiltration of the intestinal wall causes a combination of hormone receptor alteration and neuromuscular disorders that commonly result in reduced peristalsis presenting as constipation [10]. Deposition of mucopolysaccharides in the bowel wall separates muscle fibers from the ganglia, worsening autonomic neuropathy and resulting in dilatation and loss of elasticity [10]. Decreased thyroid hormone also results in decreased stimulation of the sympathetic nervous system signaling in the gastrointestinal tract, which contributes to decreased gut motility [4]. A small subset of rare, severe cases of hypothyroidism have resulted in ileus and pseudo-obstruction that, if misdiagnosed, may lead to harmful or deadly surgical intervention [11]. Though rare, it may be valuable to include hypothyroidism in the differential diagnosis for underlying conditions causing functional SBO to avoid these outcomes, and because treatment with thyroid hormone therapy has been shown to alleviate the symptoms of decreased gut motility [4].

Appropriately assessing the signs and symptoms of ileus or pseudo-obstruction to account for hypothyroidism in the differential diagnosis requires a thorough history and examination in addition to the expected imaging component. As with this patient, it should also occur in the absence of more common etiologies for bowel obstruction. Given the lack of overt intraoperative findings and an inflammatory bowel disease workup presumed to be negative, hypothyroidism offered one of the only etiological explanations for this patient’s presentation. This patient’s histopathology staining revealing mesenteric fibroadipose tissue with pericellular fibrosis and septal fibrosis could be consistent with hypothyroidism, as the disease process has been shown to cause mesenteric adipocyte dysfunction in the rat model [12]. Other cases of hypothyroid adynamic ileus in the literature are marked by important details, such as a family history of unspecified thyroiditis [13], co-presentation with other disease processes such as Hashimoto’s encephalopathy [14], concurrent infection [15], or polyneuropathy of multiple systems (intestines, bladder, and skeletal muscles) [16]. However, a seemingly equal number of cases presented with solely gastrointestinal effects [17,18]. Similar to less severe gut dysmotility caused by hypothyroidism, thyroid hormone replacement therapy improved symptoms, and patients regained normal function after consistent thyroid hormone replacement and supportive care in all reported cases of hypothyroidism-associated adynamic ileus and pseudo-obstruction [17,18].

## Conclusions

Though this patient eventually improved with levothyroxine, this case presented a diagnostic management dilemma as surgery was performed. Given the acuity and severity of this patient’s symptoms, coupled with the feces sign on his preoperative CT imaging, mechanical SBO could not be ruled out at that time. For patients with mechanical SBO, surgery is simultaneously diagnostic and therapeutic, as obstructions can be identified and addressed. This patient’s intraoperative findings were suggestive of adynamic ileus, and his clinical improvement throughout his hospital stay confirmed that diagnosis. The hypothesis that the patient’s adynamic ileus was associated with his hypothyroidism represents a clinical suspicion, as no other reasonable explanation could be offered.

While adynamic ileus as the gastrointestinal manifestation of hypothyroidism remains rare, it should be considered in the differential diagnosis, especially in cases with no prior surgical history and non-specific intraoperative findings. Further, educating hypothyroidism patients about the signs of adynamic ileus may help avoid such severe complications and encourage therapy adherence.
